# Hydroxonium 1-ammonio­ethane-1,1-diyl­diphospho­nate

**DOI:** 10.1107/S1600536809008770

**Published:** 2009-03-19

**Authors:** Ming Li, Wen Wen, Wuzu Ha, Liang Chang

**Affiliations:** a Department of Chemical Engineering, Wuhan University of Science and Engineering, Wuhan 430073, People’s Republic of China

## Abstract

The title complex, H_3_O^+^·NH_3_C(CH_3_)(PO_3_H)_2_^−^, contains a hydroxonium ion and an NH_3_C(CH_3_)(PO_3_H)_2_^−^ anion. The three H atoms of H_3_O^+^ form a pseudo-tetra­hedron by being distributed over four positions with occupation factors of 0.75. Multiple N—H⋯O and O—H⋯O hydrogen bonds in the crystal structure form an intricate three-dimensional supra­molecular network.

## Related literature

For the structures of organophospho­nates, see: Clearfield (2002 ▶); Finn *et al.* (2003 ▶). For similar bis­phospho­nates, see: Fernández *et al.* (2003 ▶); For complexes with 1-amino­ethyl­idene-1,1-diphospho­nic acid, see: Yin *et al.* (2005 ▶); Ding *et al.* (2006 ▶); Li *et al.* (2008 ▶). For the synthesis, see: Chai *et al.* (1980 ▶).
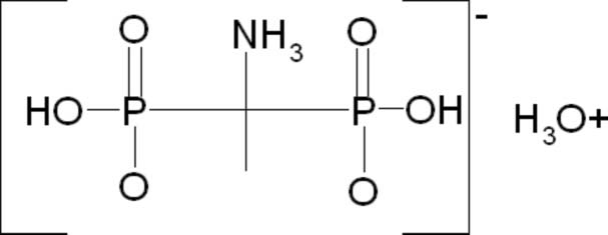


## Experimental

### 

#### Crystal data

H_3_O^+^·C_2_H_8_N_2_O_6_P_2_^−^
*M* *_r_* = 223.06
Monoclinic, 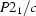

*a* = 7.3372 (6) Å
*b* = 10.6553 (8) Å
*c* = 10.6128 (8) Å
β = 97.705 (1)°
*V* = 822.22 (11) Å^3^
*Z* = 4
Mo *K*α radiation
μ = 0.53 mm^−1^
*T* = 293 K
0.36 × 0.27 × 0.18 mm

#### Data collection

Bruker SMART 4K CCD area-detector diffractometer
Absorption correction: multi-scan (*SADABS*; Sheldrick, 2008*a* ▶) *T*_min_ = 0.831, *T*_max_ = 0.910
5340 measured reflections
1972 independent reflections
1837 reflections with *I* > 2σ(*I*)
*R*_int_ = 0.015

#### Refinement

*R*[*F* ^2^ > 2σ(*F* ^2^)] = 0.032
*wR*(*F* ^2^) = 0.097
*S* = 1.10
1972 reflections
136 parameters
4 restraints
H atoms treated by a mixture of independent and constrained refinement
Δρ_max_ = 0.40 e Å^−3^
Δρ_min_ = −0.61 e Å^−3^

### 

Data collection: *SMART* (Bruker, 2001 ▶); cell refinement: *SAINT* (Bruker, 2001 ▶); data reduction: *SAINT*; program(s) used to solve structure: *SHELXS97* (Sheldrick, 2008*b* ▶); program(s) used to refine structure: *SHELXL97* (Sheldrick, 2008*b* ▶); molecular graphics: *ORTEP-3 for Windows* (Farrugia, 1997 ▶) and *PLATON* (Spek, 2009 ▶); software used to prepare material for publication: *SHELXL97*.

## Supplementary Material

Crystal structure: contains datablocks I, global. DOI: 10.1107/S1600536809008770/rn2053sup1.cif

Structure factors: contains datablocks I. DOI: 10.1107/S1600536809008770/rn2053Isup2.hkl

Additional supplementary materials:  crystallographic information; 3D view; checkCIF report

## Figures and Tables

**Table 1:** Hydrogen-bond geometry (Å, °)

*D*—H⋯*A*	*D*—H	H⋯*A*	*D*⋯*A*	*D*—H⋯*A*
N1—H1*C*⋯O2^i^	0.89	1.98	2.809 (2)	155
N1—H1*A*⋯O5^i^	0.89	1.90	2.713 (2)	151
N1—H1*B*⋯O3^ii^	0.89	2.01	2.824 (2)	152
O4—H3⋯O3^ii^	0.73 (4)	1.86 (4)	2.591 (2)	176 (4)
O1—H4⋯O6^iii^	0.71 (3)	1.84 (3)	2.550 (2)	172 (4)
O1*W*—H5⋯O5^iv^	0.893 (10)	1.954 (15)	2.804 (2)	159 (3)
O1*W*—H8⋯O1^v^	0.888 (10)	2.64 (4)	3.061 (2)	110 (3)
O1*W*—H8⋯O3^vi^	0.888 (10)	2.27 (2)	3.041 (2)	145 (3)
O1*W*—H6⋯O2^vii^	0.896 (10)	1.935 (11)	2.828 (2)	175 (3)
O1*W*—H7⋯O6^iii^	0.900 (10)	1.920 (11)	2.815 (2)	173 (3)

Symmetry codes: (i) 
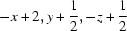
; (ii) 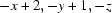; (iii) 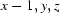; (iv) 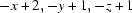; (v) 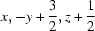; (vi) 
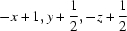
; (vii) 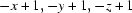.

**Fig. 1.**
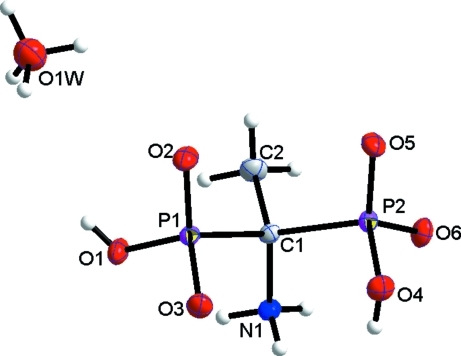
The asymmetric unit of (I) with the atom-labelling scheme. Displacement ellipsoids are drawn at the 30% probability level.

**Fig. 2.**
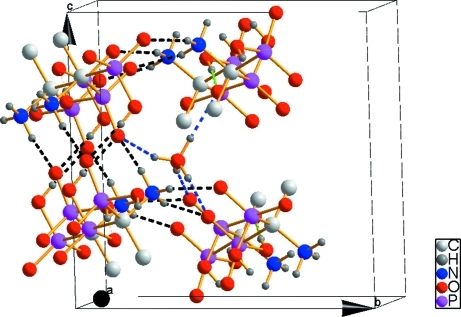
Partial packing view of compound ( I ), showing the formation of the three dimensional network built from hydrogen bonds. For the sake of clarity, H atoms not involved in hydrogen bonding have been omitted.

## supplementary crystallographic information

### Comment

Organophosphonic acids and their compounds have attracted tremendous interest. A series of phosphonate hybrid materials have been prepared and show potential applications in catalysts, sensors, sorbents, magnetic and luminescent materials. Such materials also illustrate a variety of structures from one-dimensional chains, two-dimensional layers to three-dimensional porous frameworks. (Finn *et al.*, 2003). Introduction of some functional groups to phosphonic acids, such as crown ether, –COOH, –OH, –NR_2_ or mixed groups will modify their complexing ability and construct a great number of novel phosphonates (Clearfield, 2002). Compared with other phosphonic acids, 1-aminoethylidene-1,1-diphosphonic acid (AEDPH_4_) is easier to synthesize. However, little attention has been paid to the structural study of metal-AEDP compounds (Yin *et al.*, 2005; Ding *et al.*, 2006). In our recent paper, it is found that AEDPH_4_ is inclined to transfer one proton to the amino group, which is in agreement with Fernández's results on similar bisphosphonates. (Li *et al.*, 2008; Fernández *et al.*, 2003). Deprotonation of it will result in predictable hydrogen aggregates from stronger P—O—H···O—P to weaker C—H···O hydrogen bonds. Herein, we report its structure, (I).

The asymmetric unit of (I)is built up from one deprotonated AEDPH_3_ anion and a disordered H_3_O^+^ cation, which are linked through four types of Ow-H···O hydrogen bonds (Fig. 1, Table 1). Two of the four protons of phosphonates are used in protonation, one for the amino group, the other for the H_3_O^+^ cation. The combination of different hydrogen bond interactions, N-H···O and O-H···O results in the formation of an intricate three dimensional supramolecular network (Fig.2, Table 1).

### Experimental

The AEDPH_4_ was synthesized according to the US Patent 4239695 (Chai *et al.*, 1980). It was crystallized directly from the AEDPH_4_ aqueous solution. When the mixture was heated for 24h, colorless crystals were obtained.

### Refinement

All H atoms attached to C and N atoms were fixed geometrically and treated as riding with C—H = 0.96 Å (C), N—H = 0.89Å with U_iso_(H) = 1.5U_eq_(C,N). The H atoms of hydroxyl were located in difference Fourier maps and included in the subsequent refinement.

The three hydrogen atoms of the H_3_O^+^ cation are statistically distributed over four positions with occupation factor of 0.75, building a pseudo tetrahedron.

### Crystal data

**Table:**

H_3_O^+^·C_2_H_8_N_2_O_6_P_2_^−^	*F*(000) = 464
*M**_r_* = 223.06	*D*_x_ = 1.802 Mg m^−^^3^
Monoclinic, *P*2_1_/*c*	Mo *K*α radiation, λ = 0.71073 Å
Hall symbol: -P 2ybc	Cell parameters from 3640 reflections
*a* = 7.3372 (6) Å	θ = 2.7–29.8°
*b* = 10.6553 (8) Å	µ = 0.53 mm^−^^1^
*c* = 10.6128 (8) Å	*T* = 293 K
β = 97.705 (1)°	Plate, colorless
*V* = 822.22 (11) Å^3^	0.36 × 0.27 × 0.18 mm
*Z* = 4	

### Data collection

**Table:**

Bruker SMART 4K CCD area-detector diffractometer	1837 reflections with *I* > 2σ(*I*)
graphite	*R*_int_ = 0.015
φ and ω scans	θ_max_ = 28.0°, θ_min_ = 2.7°
Absorption correction: multi-scan (*SADABS*; Sheldrick, 2008a)	*h* = −9→9
*T*_min_ = 0.831, *T*_max_ = 0.910	*k* = −9→14
5340 measured reflections	*l* = −13→11
1972 independent reflections	

### Refinement

**Table:**

Refinement on *F*^2^	Secondary atom site location: difference Fourier map
Least-squares matrix: full	Hydrogen site location: inferred from neighbouring sites
*R*[*F*^2^ > 2σ(*F*^2^)] = 0.032	H atoms treated by a mixture of independent and constrained refinement
*wR*(*F*^2^) = 0.097	*w* = 1/[σ^2^(*F*_o_^2^) + (0.0481*P*)^2^ + 0.8715*P*] where *P* = (*F*_o_^2^ + 2*F*_c_^2^)/3
*S* = 1.10	(Δ/σ)_max_ = 0.001
1972 reflections	Δρ_max_ = 0.40 e Å^−^^3^
136 parameters	Δρ_min_ = −0.61 e Å^−^^3^
4 restraints	Extinction correction: *SHELXL97* (Sheldrick, 2008), Fc^*^=kFc[1+0.001xFc^2^λ^3^/sin(2θ)]^-1/4^
Primary atom site location: structure-invariant direct methods	Extinction coefficient: 0.023 (2)

### Special details

**Table:**

Geometry. All esds (except the esd in the dihedral angle between two l.s. planes) are estimated using the full covariance matrix. The cell esds are taken into account individually in the estimation of esds in distances, angles and torsion angles; correlations between esds in cell parameters are only used when they are defined by crystal symmetry. An approximate (isotropic) treatment of cell esds is used for estimating esds involving l.s. planes.

### Fractional atomic coordinates and isotropic or equivalent isotropic displacement parameters (Å^2^)

**Table:**

	*x*	*y*	*z*	*U*_iso_*/*U*_eq_	Occ. (<1)
C1	0.9948 (2)	0.56114 (16)	0.24265 (16)	0.0131 (3)	
C2	0.9672 (3)	0.62357 (19)	0.36861 (18)	0.0209 (4)	
H2A	1.0741	0.6722	0.3993	0.031*	
H2B	0.9488	0.5602	0.4299	0.031*	
H2C	0.8615	0.6774	0.3556	0.031*	
N1	1.0165 (2)	0.66564 (14)	0.14939 (14)	0.0152 (3)	
H1A	0.9151	0.7124	0.1391	0.023*	
H1B	1.0347	0.6329	0.0750	0.023*	
H1C	1.1124	0.7131	0.1792	0.023*	
O1W	0.4462 (2)	0.70203 (18)	0.52441 (17)	0.0363 (4)	
O1	0.63612 (19)	0.57626 (13)	0.17124 (14)	0.0209 (3)	
O2	0.75556 (18)	0.37252 (13)	0.27913 (13)	0.0208 (3)	
O3	0.80235 (18)	0.42568 (13)	0.05066 (12)	0.0208 (3)	
O4	1.23166 (19)	0.41015 (14)	0.13108 (14)	0.0209 (3)	
O5	1.20389 (18)	0.37077 (13)	0.35944 (13)	0.0204 (3)	
O6	1.36080 (17)	0.56986 (13)	0.29441 (13)	0.0199 (3)	
P1	0.78594 (6)	0.47061 (4)	0.18354 (4)	0.01339 (15)	
P2	1.21301 (6)	0.47175 (4)	0.26319 (4)	0.01358 (15)	
H3	1.227 (5)	0.456 (3)	0.079 (3)	0.051 (10)*	
H4	0.562 (5)	0.568 (3)	0.208 (3)	0.049 (10)*	
H5	0.5658 (17)	0.698 (3)	0.554 (3)	0.018 (7)*	0.75
H6	0.386 (4)	0.673 (3)	0.587 (2)	0.018 (7)*	0.75
H7	0.426 (4)	0.663 (2)	0.4485 (15)	0.013 (7)*	0.75
H8	0.422 (5)	0.7827 (13)	0.509 (4)	0.038 (10)*	0.75

### Atomic displacement parameters (Å^2^)

**Table:**

	*U*^11^	*U*^22^	*U*^33^	*U*^12^	*U*^13^	*U*^23^
C1	0.0145 (7)	0.0114 (7)	0.0138 (7)	−0.0006 (6)	0.0032 (6)	0.0007 (6)
C2	0.0254 (9)	0.0214 (9)	0.0164 (8)	0.0030 (7)	0.0042 (7)	−0.0040 (7)
N1	0.0165 (7)	0.0123 (7)	0.0173 (7)	−0.0002 (5)	0.0040 (5)	0.0017 (5)
O1W	0.0356 (9)	0.0393 (10)	0.0331 (9)	0.0008 (8)	0.0010 (7)	−0.0001 (7)
O1	0.0155 (6)	0.0200 (7)	0.0283 (7)	0.0043 (5)	0.0073 (5)	0.0065 (5)
O2	0.0210 (6)	0.0164 (6)	0.0261 (7)	0.0001 (5)	0.0068 (5)	0.0073 (5)
O3	0.0217 (6)	0.0236 (7)	0.0169 (6)	−0.0005 (5)	0.0022 (5)	−0.0037 (5)
O4	0.0248 (7)	0.0184 (7)	0.0201 (7)	0.0019 (5)	0.0051 (5)	−0.0036 (5)
O5	0.0191 (6)	0.0189 (6)	0.0227 (7)	0.0010 (5)	0.0005 (5)	0.0066 (5)
O6	0.0148 (6)	0.0193 (6)	0.0259 (7)	−0.0038 (5)	0.0037 (5)	−0.0049 (5)
P1	0.0127 (2)	0.0124 (2)	0.0152 (2)	−0.00029 (15)	0.00251 (16)	0.00126 (15)
P2	0.0123 (2)	0.0125 (2)	0.0158 (2)	0.00017 (15)	0.00167 (16)	0.00006 (15)

### Geometric parameters (Å, °)

**Table:**

C1—N1	1.512 (2)	O1W—H6	0.896 (10)
C1—C2	1.531 (2)	O1W—H7	0.900 (10)
C1—P1	1.8479 (17)	O1W—H8	0.888 (10)
C1—P2	1.8505 (17)	O1—P1	1.5666 (14)
C2—H2A	0.9600	O1—H4	0.71 (3)
C2—H2B	0.9600	O2—P1	1.4940 (13)
C2—H2C	0.9600	O3—P1	1.5093 (13)
N1—H1A	0.8900	O4—P2	1.5706 (14)
N1—H1B	0.8900	O4—H3	0.73 (4)
N1—H1C	0.8900	O5—P2	1.4914 (13)
O1W—H5	0.893 (10)	O6—P2	1.5106 (13)
			
N1—C1—C2	106.82 (14)	H5—O1W—H7	109 (3)
N1—C1—P1	108.51 (11)	H6—O1W—H7	118 (3)
C2—C1—P1	108.82 (12)	H5—O1W—H8	106 (3)
N1—C1—P2	106.91 (11)	H6—O1W—H8	111 (3)
C2—C1—P2	109.54 (12)	H7—O1W—H8	106 (3)
P1—C1—P2	115.87 (9)	P1—O1—H4	116 (3)
C1—C2—H2A	109.5	P2—O4—H3	113 (3)
C1—C2—H2B	109.5	O2—P1—O3	116.77 (8)
H2A—C2—H2B	109.5	O2—P1—O1	113.10 (8)
C1—C2—H2C	109.5	O3—P1—O1	107.04 (8)
H2A—C2—H2C	109.5	O2—P1—C1	109.10 (8)
H2B—C2—H2C	109.5	O3—P1—C1	108.44 (8)
C1—N1—H1A	109.5	O1—P1—C1	101.18 (8)
C1—N1—H1B	109.5	O5—P2—O6	116.47 (8)
H1A—N1—H1B	109.5	O5—P2—O4	109.10 (8)
C1—N1—H1C	109.5	O6—P2—O4	109.82 (8)
H1A—N1—H1C	109.5	O5—P2—C1	109.54 (8)
H1B—N1—H1C	109.5	O6—P2—C1	104.71 (8)
H5—O1W—H6	107 (3)	O4—P2—C1	106.71 (8)
			
N1—C1—P1—O2	−176.13 (11)	N1—C1—P2—O5	177.18 (11)
C2—C1—P1—O2	−60.25 (14)	C2—C1—P2—O5	61.79 (14)
P2—C1—P1—O2	63.66 (11)	P1—C1—P2—O5	−61.75 (11)
N1—C1—P1—O3	55.70 (13)	N1—C1—P2—O6	51.57 (12)
C2—C1—P1—O3	171.58 (12)	C2—C1—P2—O6	−63.82 (13)
P2—C1—P1—O3	−64.51 (11)	P1—C1—P2—O6	172.64 (9)
N1—C1—P1—O1	−56.68 (12)	N1—C1—P2—O4	−64.85 (12)
C2—C1—P1—O1	59.20 (13)	C2—C1—P2—O4	179.76 (12)
P2—C1—P1—O1	−176.89 (9)	P1—C1—P2—O4	56.22 (11)

### Hydrogen-bond geometry (Å, °)

**Table:**

*D*—H···*A*	*D*—H	H···*A*	*D*···*A*	*D*—H···*A*
N1—H1C···O2^i^	0.89	1.98	2.809 (2)	155
N1—H1A···O5^i^	0.89	1.90	2.713 (2)	151
N1—H1B···O3^ii^	0.89	2.01	2.824 (2)	152
O4—H3···O3^ii^	0.73 (4)	1.86 (4)	2.591 (2)	176 (4)
O1—H4···O6^iii^	0.71 (3)	1.84 (3)	2.550 (2)	172 (4)
O1W—H5···O5^iv^	0.89 (1)	1.95 (2)	2.804 (2)	159 (3)
O1W—H8···O1^v^	0.89 (1)	2.64 (4)	3.061 (2)	110 (3)
O1W—H8···O3^vi^	0.89 (1)	2.27 (2)	3.041 (2)	145 (3)
O1W—H6···O2^vii^	0.90 (1)	1.94 (1)	2.828 (2)	175 (3)
O1W—H7···O6^iii^	0.90 (1)	1.92 (1)	2.815 (2)	173 (3)

Symmetry codes: (i) −*x*+2, *y*+1/2, −*z*+1/2; (ii) −*x*+2, −*y*+1, −*z*; (iii) *x*−1, *y*, *z*; (iv) −*x*+2, −*y*+1, −*z*+1; (v) *x*, −*y*+3/2, *z*+1/2; (vi) −*x*+1, *y*+1/2, −*z*+1/2; (vii) −*x*+1, −*y*+1, −*z*+1.
